# Low-Temperature Plasma-Enhanced Atomic Layer Deposition of SiO_2_ Using Carbon Dioxide

**DOI:** 10.1186/s11671-019-2889-y

**Published:** 2019-02-12

**Authors:** Zhen Zhu, Perttu Sippola, Oili M. E. Ylivaara, Chiara Modanese, Marisa Di Sabatino, Kenichiro Mizohata, Saoussen Merdes, Harri Lipsanen, Hele Savin

**Affiliations:** 1 Department of Electronics and Nanoengineering, Aalto University, Tietotie 3, FI-02150 Espoo, Finland; 2 Beneq Oy, Olarinluoma 9, FI-02200 Espoo, Finland; 3 VTT Technical Research Centre of Finland Ltd., P. O. Box 1000, FI-02044 VTT Espoo, Finland; 4 Department of Materials Science and Engineering, Norwegian University of Science and Technology (NTNU), Alfred Getz vei 2B, 7491 Trondheim, Norway; 5 Division of Materials Physics, Physics Department, University of Helsinki, Gustaf Hällströmin katu 2a, FI-00014 Helsinki, Finland

**Keywords:** Carbon dioxide, Silicon dioxide, ALD, Plasma, Radicals, Oxidation

## Abstract

In this work, we report the successful growth of high-quality SiO_2_ films by low-temperature plasma-enhanced atomic layer deposition using an oxidant which is compatible with moisture/oxygen sensitive materials. The SiO_2_ films were grown at 90 °C using CO_2_ and Bis(tertiary-butylamino)silane as process precursors. Growth, chemical composition, density, optical properties, and residual stress of SiO_2_ films were investigated. SiO_2_ films having a saturated growth-per-cycle of ~ 1.15 Å/cycle showed a density of ~ 2.1 g/cm^3^, a refractive index of ~ 1.46 at a wavelength of 632 nm, and a low tensile residual stress of ~ 30 MPa. Furthermore, the films showed low impurity levels with bulk concentrations of ~ 2.4 and ~ 0.17 at. % for hydrogen and nitrogen, respectively, whereas the carbon content was found to be below the measurement limit of time-of-flight elastic recoil detection analysis. These results demonstrate that CO_2_ is a promising oxidizing precursor for moisture/oxygen sensitive materials related plasma-enhanced atomic layer deposition processes.

## Background

SiO_2_ is a widely used material for applications such as microelectronics [1, 2], microelectromechanical systems [3, 4], photovoltaics [5, 6], and optics [7, 8]. While SiO_2_ thin films can be grown by several methods such as thermal oxidation, plasma-enhanced chemical vapor deposition (PECVD), or physical vapor deposition (PVD), atomic layer deposition (ALD) offers the exceptional advantage of combining precise film thickness control, high uniformity, and conformality [9–11].

Many ALD processes, with various Si precursors (chlorosilanes or aminosilanes) and oxidants (H_2_O, H_2_O_2_, or O_3_), were developed for the growth of SiO_2_. Those processes usually require relatively high temperatures (> 150 °C) [12–16]. For processes compatible with thermally sensitive materials such as organic, biological, and polymeric materials, the catalyzed ALD [17–19] and plasma-enhanced atomic layer deposition (PEALD) [9, 20–22] have been used as an effective solution with process temperatures below 100 °C. However, the commonly used H_2_O and O_2_-based oxidants can lead to material degradation in the case of moisture/oxygen sensitive materials. Compared to H_2_O and O_2_, at low-temperature, CO_2_ is not chemically reactive. In this case, using CO_2_ as an oxidant can minimize the degradation of moisture/oxygen sensitive materials by avoiding unnecessary oxidization. Furthermore, CO_2_ was reported by King [23] to be a viable oxidizing agent for the growth of PEALD SiO_2_ films when using SiH_4_ as a Si precursor. However, the growth temperatures of those PEALD processes, which were in the range of 250–400 °C, are not compatible with high-temperature sensitive materials.

In this work, we report the development of a CO_2_-based PEALD process for SiO_2_ films at 90 °C. The dependence of the film growth on the process parameters (precursor pulse/purge time and plasma power) is investigated. We also report the chemical composition, structural and optical properties, and residual stress analysis of the films.

## Methods

### Film Preparation

PEALD SiO_2_ films were grown on Si(100) and sapphire substrates at 90 °C using CO_2_ (99.5%, Air Products) plasma as oxygen source and bis(tertiary-butylamino)silane (BTBAS) (97%, Strem Chemicals) as Si precursor [22]. The processes were carried out in a Beneq TFS 200 reactor with a remote plasma system using a capacitively coupled 13.56 MHz radio frequency (rf)-operated source. The N_2_ (99.999%, AGA) was used as a carrier and purge gas with a through-reactor flow of 600 sccm, while a mixture gas of N_2_ (200 sccm) and CO_2_ (75 sccm) flowed through the plasma system. The chosen plasma powers in this study were based on the stability of the plasma system. The source temperature of BTBAS was set at 21 °C and a N_2_ booster was applied during precursor pulse. The details of process parameters are shown in Table 1. During the PEALD processes, the reactor pressure was about 1 hPa.

**Table 1:** The main parameters of the PEALD process

Growth parameter	Range
Process temperature (°C)	90
Plasma power (W)	50–300
BTBAS pulse time (s)	0.05–0.5
BTBAS purge time (s)	0.5–3
CO_2_ plasma exposure time (s)	1–15
CO_2_ plasma purge time (s)	0.5–3

### Film Characterization

The thickness of PEALD SiO_2_ films was determined with a SENTECH SE400adv ellipsometer using a HeNe laser at a wavelength of 632.8 nm and at an incident angle of 70°. The growth-per-cycle (GPC) was calculated using the obtained film thickness divided by the number of ALD cycles. The deviation of the GPC was based on the non-uniformity of film thickness.

Chemical composition was measured by glow-discharge optical emission spectroscopy (GDOES), time-of-flight elastic recoil detection analysis (TOF-ERDA), and attenuated total reflectance Fourier transform infrared spectroscopy (ATR-FTIR). GDOES measurements were carried out on a Horiba GD-Profiler 2. A 4-mm-diameter anode and rf power of 35 W in the pulsed mode were used. The elemental intensities were reported as values integrated over the whole film thickness as described in Ref. [22]. For TOF-ERDA measurements, 40 MeV energy Br ions obtained from a 5MV tandem accelerator were directed on the measured samples. The detection angle was 40°. ATR-FTIR measurements were done using a Thermo Electron Corporation Nicolet 380 ATR-FTIR spectrometer with a diamond crystal as an internal reflection element. The procedure included a background collection from the Si substrate and data collection from the samples. A 2-cm^−1^ resolution over the 800–4000 cm^−1^ wavenumber range was used.

X-ray reflectivity (XRR) analyses were performed with a Philips X’Pert Pro diffractometer using Cu-K_α1_ radiation. The film density was acquired from the measured data by using an in-house-developed fitting software [24]. An interfacial oxide layer between the silicon substrate and the PEALD SiO_2_ film was simulated as a part of the XRR fitting layer model. Using a PerkinElmer Lambda 900 spectrometer, transmittance spectrum of the PEALD SiO_2_ film was recorded in the 360–800 nm wavelength range following the growth on sapphire substrate. The refractive index (*n*) and extinction coefficient (*k*) were determined with Cauchy fitting from the transmittance spectrum. To ensure good fitting accuracy, for this measurement, 150-nm-thick SiO_2_ films were grown on sapphire substrates.

The residual stress of 50-nm-thick PEALD SiO_2_ films was determined with the wafer curvature method [25] and Stoney’s equation [26]. The wafer curvature was measured before and after film growth with a TOHO FLX-2320-S tool. The wafers were scanned biaxially using a 120-mm scan length. Measured results were presented with maximum measurement uncertainty [25].

## Results and Discussion

### Film Growth

**Fig. 1:**
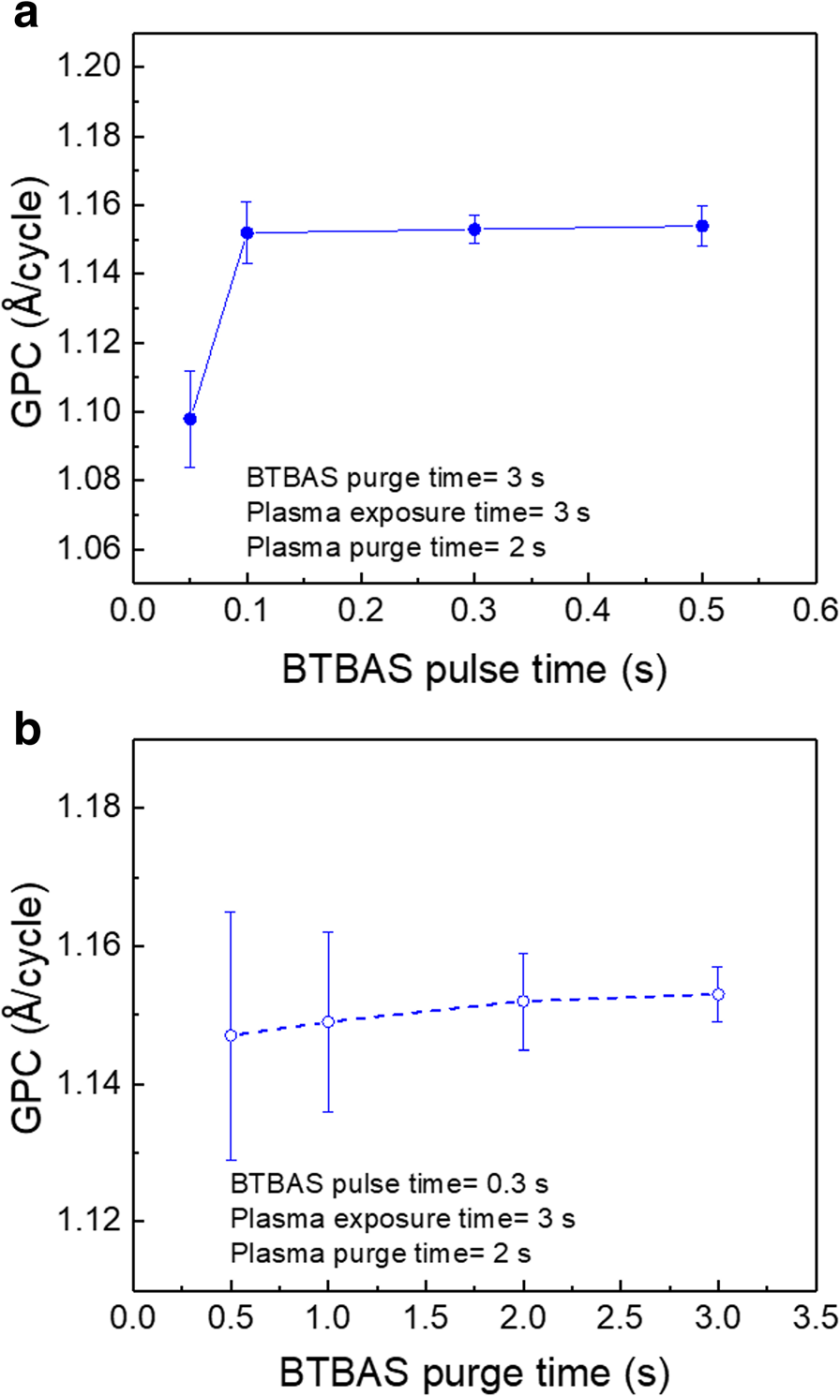
GPC of PEALD SiO_2_ films grown on Si substrates as a function of BTBAS **a** pulse time and **b** purge time. The applied plasma power was 180 W

The dependence of the SiO_2_ film GPC on the BTBAS pulse and purge time was investigated during the oxidation step with a fixed plasma power of 180 W, a CO_2_ plasma exposure time of 3 s, and a CO_2_ plasma purge time of 2 s. Figure 1a and b show GPC values as a function of BTBAS pulse and purge time, respectively. For the dependence on pulse time, the BTBAS purge time was set to 3 s, whereas for the dependence on purge time, the BTBAS pulse time was set to 0.3 s. As shown in Fig. 1a, the lowest GPC is obtained with a BTBAS pulse of 0.05 s, while a pulse time of 0.1 s is found to be sufficient to reach a self-limiting growth with a GPC of ~ 1.15 Å/cycle. Moreover, when a fixed BTBAS pulse of 0.3 s and a decreasing purge time from 3 to 0.5 s (Fig. 1b) are used, no change of GPC is observed. This indicates that the applied short purge time of BTBAS is sufficient to prevent CVD components. Note however that the uniformity of film thickness was improved with increasing purge time.

**Fig. 2:**
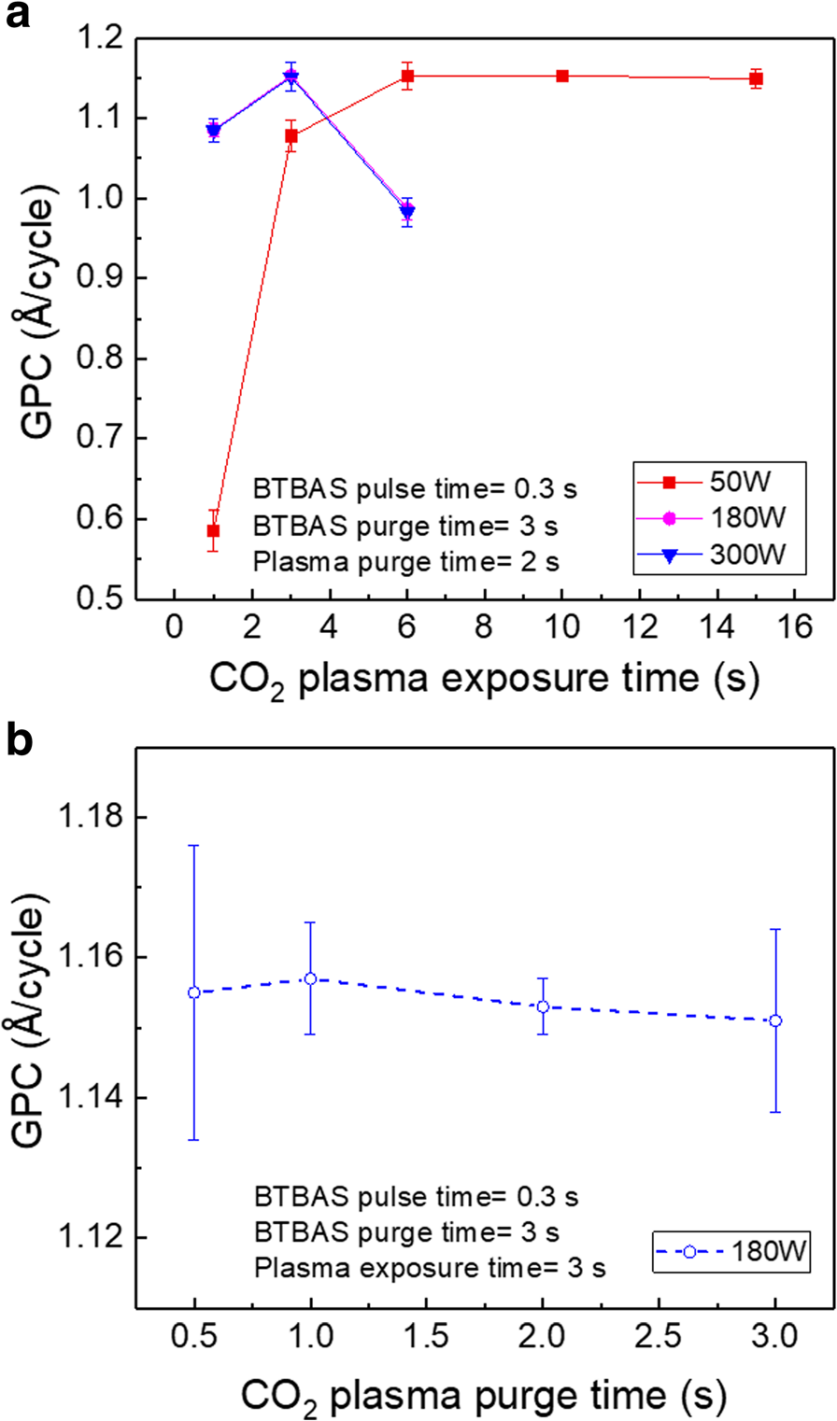
GPC of PEALD SiO_2_ films grown on Si substrates as a function of CO_2_ plasma **a** exposure time with varied plasma powers of 50, 180, and 300 W, and **b** purge time with a plasma power of 180 W

The SiO_2_ growth during the oxidation step was investigated using fixed BTBAS pulse and purge time of 0.3 and 3 s, respectively. Figure 2a and b show the GPC of PEALD SiO_2_ films grown on Si wafers as a function of the CO_2_ plasma exposure and purge time, respectively. During the study of plasma exposure time effects, plasma powers of 50, 180, and 300 W were applied. As a general observation, the highest GPC value of 1.15 Å/cycle, which can be considered as the saturated GPC at 90 °C [27], is observed in all plasma power conditions. For the process with a plasma exposure time of 1 s, independently of the used power, GPC values below 1.15 Å/cycle hint to incomplete film growth. This indicates that the plasma exposure time of 1 s is not long enough to generate a sufficient amount of O radicals. These radicals, which are necessary for a complete surface reaction, result from CO_2_ plasma dissociation reactions [28]. For a plasma power of 50 W, the GPC is found to increase with an increase of CO_2_ plasma exposure time until 6 s, after which the GPC reaches the saturated value of 1.15 Å/cycle. This value remains constant for an exposure time up to 15 s. However, for films grown with higher power (180 and 300 W), an inverse V trend of the GPC is observed for plasma exposure time between 1 and 6 s. According to our earlier reported growth stages of PEALD films [27] and the obtained highest GPC of 1.15 Å/cycle in this work, growth saturation is achieved at 180 and 300 W with a plasma exposure time of 3 s. For a plasma exposure time of 6 s, the decreased GPC is probably a consequence of the film densification, similar to the one we previously reported for PEALD of Al_2_O_3_ thin films [27]. Note that these two curves (depicting the dependence of the GPC on CO_2_ plasma exposure time for 180 and 300 W) fully overlap. The observed overlapping of the GPC curves suggests that the growth of the SiO_2_ films with 180 and 300 W involves identical mechanisms that could be related to a comparable amount of ion and radical fluxes generated by high power plasma [29]. Compared to the case of high powers, the growth behavior of SiO_2_ thin films using a plasma power of 50 W is different as no film densification occurs. This is most likely due to the relatively low ion and radical fluxes resulting from the low power of 50 W [29].

The effect of CO_2_ plasma purge time on the GPC is shown in Fig. 2b. As in the case of BTBAS purge time dependency, GPC values are found to remain constant when CO_2_ purge time is varied between 0.5 and 3 s. Thus, it can be concluded that the applied purge time of both precursors has a negligible impact on the GPC of our SiO_2_ thin films. This differs from an earlier reported PEALD process with SAM.24, one kindred aminosilanes of BTBAS, and O_2_ plasma [9], where purge steps with a purge time shorter than 2 s were found to have a significant effect on film growth. Here, the independence between our applied precursor purge time and the GPC could be assigned to the effective removal of residual precursors and byproducts which could partially benefit from the reaction chamber design using the cross-flow. Such configuration makes the gas exchange time between precursor pulses relatively short. Nevertheless, the stickiness of precursors cannot be ruled out. Based on the results shown in Fig. 2a, by using BTBAS pulse/purge time of 0.3 s/3 s and CO_2_ plasma exposure/purge time of 3 s/2 s, the highest deposition speed during the saturated growth is 50 nm/h. This implies that by applying a high plasma power and using BTBAS pulse/purge time of 0.1 s/0.5 s and CO_2_ plasma exposure/purge time of 3 s/0.5 s, a deposition speed of up to 100 nm/h is achievable.

### Film Properties

**Fig. 3:**
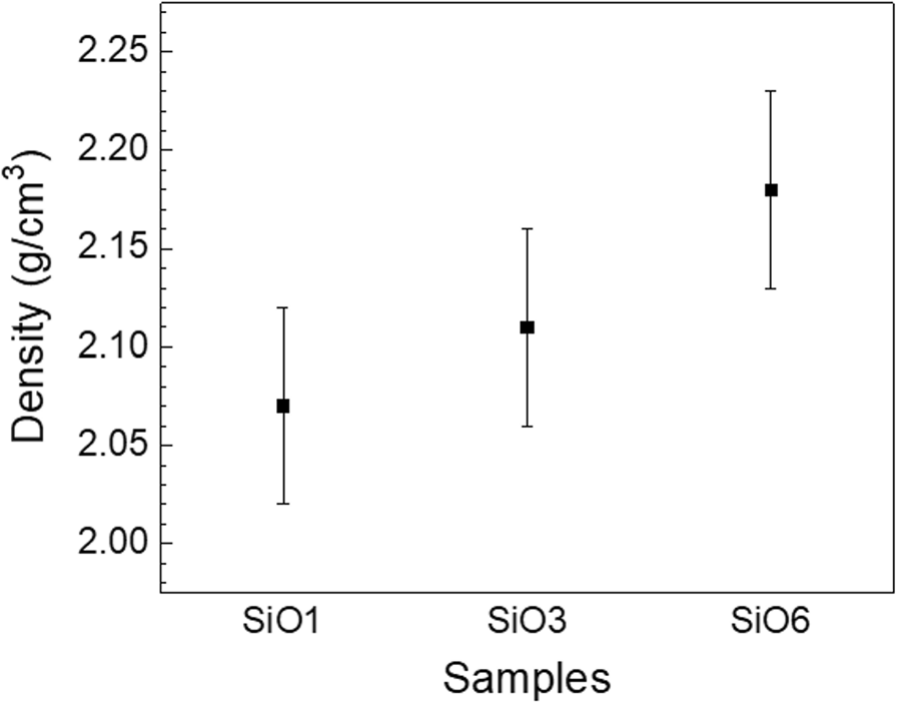
Density of SiO_2_ films grown with the plasma exposure time of 1, 3, and 6 s

The density of the SiO_2_ films was studied by XRR and the results are shown in Fig. 3. The measured samples were grown on Si substrates using varied plasma exposure time with a plasma power of 180 W, a BTBAS pulse time of 0.3 s, a BTBAS purge time of 3 s, and a CO_2_ plasma purge time of 2 s. The studied samples are labeled as “SiO1,” “SiO3,” and “SiO6” for a plasma exposure time of 1, 3, and 6 s, respectively. Although the values are within the measurement error margin, the lowest and highest mean values are shown in “SiO1” and “SiO6,” respectively, suggesting that the film density is slightly increased with an increase of plasma exposure time. This supports our hypothesis of film densification during the process with a plasma power of 180 W and an exposure time of 6 s. In the case of saturated growth, although our film density of 2.11 g/cm^3^ is in a good agreement with values reported in earlier studies for O_2_-based PEALD SiO_2_ films using commercial ALD reactors with growth temperatures ranging between 50 and 300 °C [9, 21, 30], it is lower than the value (2.3 g/cm^3^) reported by King who demonstrated the PEALD SiO_2_ process at 400 °C in a modified PECVD reactor [23].

**Fig. 4:**
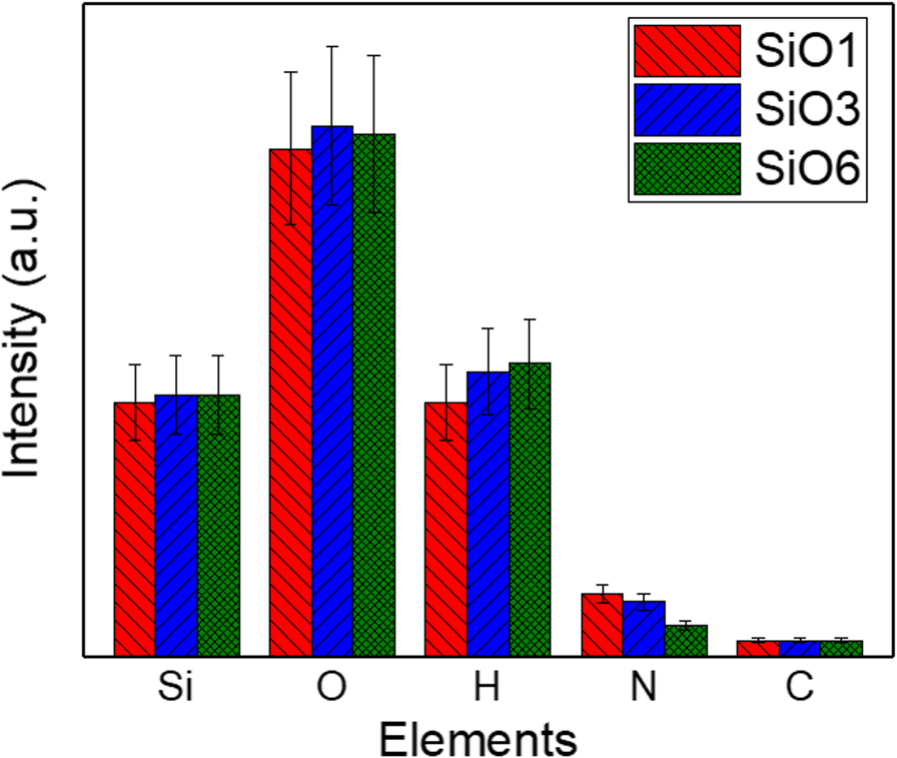
Qualitative chemical composition of SiO_2_ films grown with the plasma exposure time of 1, 3, and 6 s measured by GDOES. The measurement has an accuracy of ± 15% and the cross-elemental comparison of the intensities is not possible in this work (no calibration factor is available)

The chemical composition of PEALD SiO_2_ was measured by GDOES. Because the measurements were not calibrated for compositional depth profiling, i.e., the element-dependent emission rate was not considered, only the intensities of the same element can be compared among different samples and no comparison among different elements is possible. Therefore, in this case, the GDOES measurements provide a rather qualitative information on the chemical composition. The detected elements, Si, O, H, N, and C, are shown in Fig. 4. As displayed on the figure, although the intensity of H in “SiO1” is slightly lower than in the other samples, taking into consideration error margins, no significant effects of plasma exposure time on the Si, O, and H contents are observed. This finding is similar to plasma power effects reported in our previous work on PEALD SiO_2_ grown using BTBAS and O_2_ plasma [22]. In the case of N content, the intensities for “SiO1” and “SiO3” are rather constant, whereas a lower intensity is measured for “SiO6.” This suggests that N impurity removal is more effective during the film densification. Note that, independently of sample growth conditions, all the measured samples show the same intensity for C content.

**Fig. 5:**
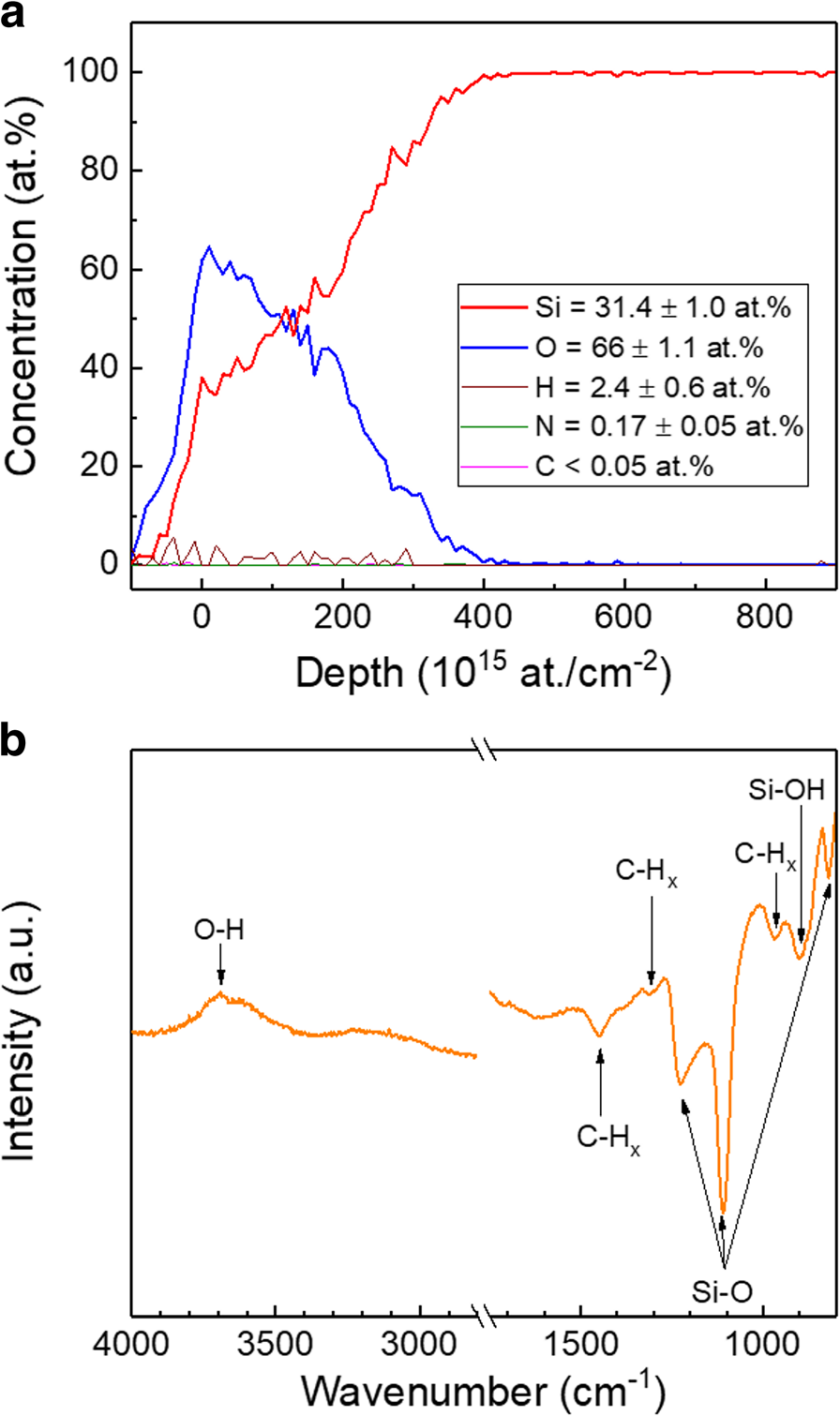
**a** TOF-ERDA depth profile and **b** ATR-FTIR transmission spectrum of the SiO_2_ film. The targeted film thickness was 50 nm

Further evaluation of film chemical composition was carried out using TOF-ERDA and ATR-FTIR measurements. Taking into account that saturated growth is normally targeted in ALD processes, in what follows, we focus our investigation on samples grown with a plasma power of 180 W, a BTBAS pulse time of 0.3 s, a BTBAS purge time of 3 s, a CO_2_ plasma exposure time of 3 s, and a CO_2_ plasma purge time of 2 s. TOF-ERDA depth profile and element composition are shown in Fig. 5a. Note that the down-slope of O shown in the film depth profile is caused by the effect of the Si substrate, which correlates to the depth resolution of TOF-ERDA for our SiO_2_/Si sample structure. During the element composition analyses, the substrate effect has been taken into consideration. The investigated sample exhibits low impurity levels with bulk concentrations of ~ 2.4 and ~ 0.17 at. % for hydrogen and nitrogen, respectively, whereas the total C concentration in whole film is found to be below the measurement limit of TOF-ERDA. Based on the information of depth profile, the carbon counts are mostly collected from the film surface. Therefore, we speculate that the C content detected by GDOES measurements and shown in Fig. 4 could be due to the sample contamination during storage or from the test environment. It is worth noting that the H concentration is also found to be higher on the surface than the bulk. Additionally, the films are found to have a slightly oxygen-rich composition with a Si/O ratio of ~ 0.48. This result is consistent with the one reported by Dingemans et al. for PEALD SiO_2_ grown using SAM.24 and O_2_ plasma in a temperature range between 100 and 300 °C [9]. This oxygen-rich composition is most likely due to the contribution of residual –OH species left to the films.

Figure 5b shows ATR-FTIR spectrum measured on the same sample. The broad band features, located in the 3200–3800 cm^−1^ region, can be assigned to the O-H stretch of the Si–OH and water but the former is less likely [14, 31]. Another band, which is also typical of the Si–OH stretch [31], is visible at ~ 900 cm^−1^. The presence of –OH groups, which is consistent with TOF-ERDA results shown above, implies that combustion-like reactions, which involve the combustion of –*NH*^*t*^*Bu* ligands and formation of –OH groups, dominate the oxidization step. A similar mechanism has been previously reported to take place during the growth of Al_2_O_3_ from trimethylaluminum and O_2_ plasma [32] and SiO_2_ from SAM.24 and O_2_ plasma [9]. In addition to the –OH groups, the Si-O-Si bond stretching is detected around 1108 and 1226 cm^−1^ [14, 33] while the bond bending is seen at approximately 820 cm^−1^ [34, 35]. Note that compared to literature values [14, 34, 35], the Si-O-Si stretching frequency in this work is relatively high. This could be caused by the change of the Si-O bond length which can be influenced by the film residual stress. Jutarosaga et al. reported that the higher the compressive stress is, the lower the Si-O-Si stretching frequency is [36]. The bands at ~ 970, 1301, and 1450 cm^−1^ are assigned to the CH_3_ rocking, CH_3_ symmetric deformation, and CH_2_ scissor, respectively [14]. The finding of C-H surface groups is in line with the result of TOF-ERDA and is most likely due to the surface contamination.

From the data in Fig. 5 and based on results previously reported in literature [37], our process surface reactions during the first ALD half-cycle can be considered as follows:

1 $$ \mathrm{Si}-{\mathrm{OH}}^{\ast }+{\mathrm{H}}_2\mathrm{Si}{\left[{NH}^t Bu\right]}_2\to \mathrm{Si}-\mathrm{O}-{\mathrm{SiH}}_2{{\left[{NH}^t Bu\right]}_{2-x}}^{\ast }+{xH}_2{N}^t Bu $$

where surface species are denoted by the asterisk (*). In the first half-reaction, only one (*x* = 1) or both (*x* = 2) of -*NH*^*t*^*Bu* ligands can react with the surface –OH groups forming *t*-butylamine molecules.

The O radicals are the main active species generated during the CO_2_ plasma dissociation reactions [28] and consequently dominate the oxidation reactions. Therefore, in the second half-cycle, the proposed combustion-like reactions [9] are:

2 $$ {\mathrm{SiH}}_2{{\left[{NH}^t Bu\right]}_{2-x}}^{\ast }+\mathrm{O}\to \mathrm{Si}-{\mathrm{OH}}^{\ast }+{\mathrm{H}}_2\mathrm{O}+{\mathrm{CO}}_2+\mathrm{N}-\mathrm{containing}\ \mathrm{species} $$

Due to the uncertainty of the actual reaction products, the proposed surface reaction is purposely not balanced. To be able to fully determine this reaction, in-situ analyses during the film growth, such as by-product gas analyses, would be needed.

**Fig. 6:**
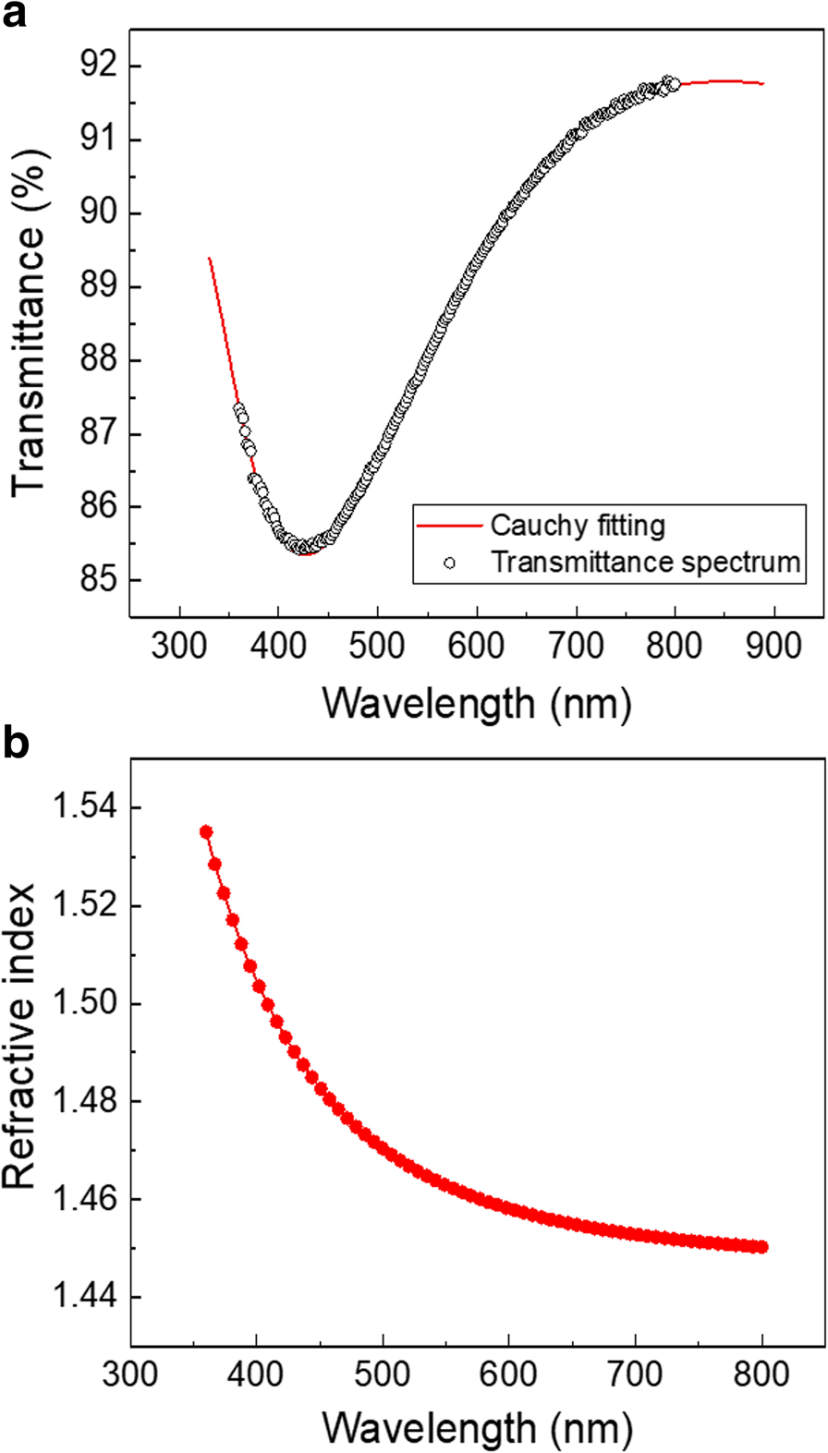
**a** Transmittance spectrum and Cauchy fitting and **b** refractive index dispersions of the SiO_2_ film grown on a sapphire substrate with a plasma power of 180 W, a BTBAS pulse time of 0.3 s, a BTBAS purge time of 3 s, a CO_2_ plasma exposure time of 3 s, and a CO_2_ plasma purge time of 2 s. Targeted film thickness was 150 nm

The optical properties of SiO_2_ film grown on a sapphire substrate were studied by spectrometry. Figure 6a shows the measured transmittance as a function of the wavelength together with the Cauchy fitting of the curve. The refractive index dispersion simulated from the transmittance spectrum is shown in Fig. 6b. From the fitting, at a wavelength of 632 nm, a refractive index of 1.456 and zero *k* value were obtained. This refractive index value is in a good agreement with what previously reported for low-temperature PEALD SiO_2_ [9, 21] and relatively low compared with values reported for high-temperature processes [23]. Indeed, growth temperature is known to influence the –OH concentration in the grown films and therefore their refractive index [38]. In addition, the obtained zero *k* value is consistent with the low carbon content in the films. A similar correlation between the *k* value and the C concentration was previously reported by Putkonen et al. for SiO_2_ thin films grown by ALD [21].

**Fig. 7:**
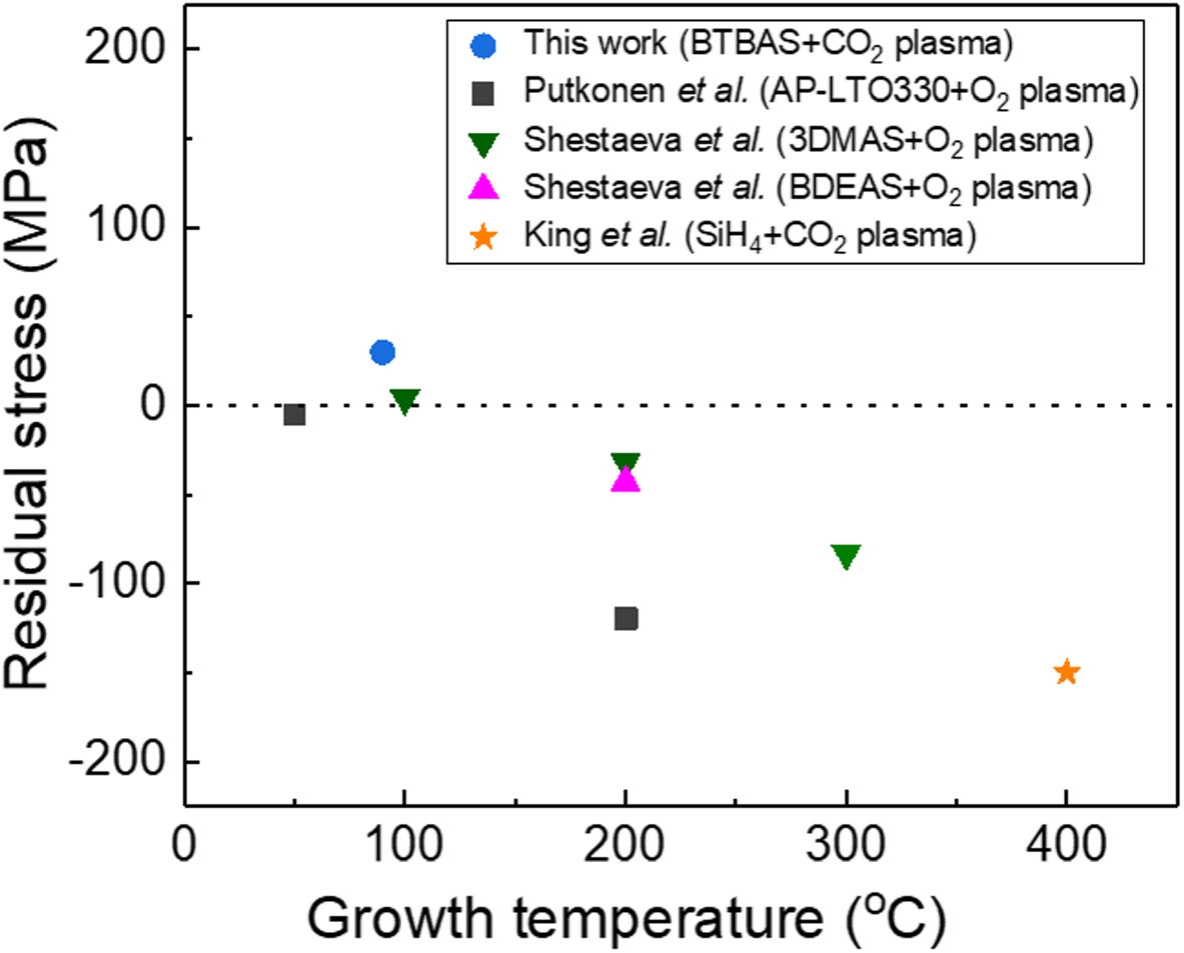
Residual stress of SiO_2_ films as a function of growth temperature. Our sample was grown with a plasma power of 180 W, a BTBAS pulse time of 0.3 s, a BTBAS purge time of 3 s, a CO_2_ plasma exposure time of 3 s, and a CO_2_ plasma purge time of 2 s. References include Putkonen et al. [21], Shestaeva et al. [30], and King [23]. Targeted film thickness of our sample was 50 nm

Residual stress of ALD films comprises the contributions of thermal stress and intrinsic stress. Thermal stress results from the difference in the thermal expansion between the film and the substrate. The intrinsic stress is defined as the internal stress created during the film growth, depending on the precursors, growth temperature, and ALD method [30, 39]. Figure 7 shows the residual stress of SiO_2_ films as a function of growth temperature. The highest stress value, 150 MPa (compressive) [23], was obtained from the sample grown at 400 °C; however, a low tensile stress of 30 ± 10 MPa is obtained at 90 °C in this work. Putkonen et al. and Shestaeva et al. showed a clear dependence of SiO_2_ film stress on growth temperature [21, 30]: higher temperature results in higher compressive stress. The contribution of thermal stress is larger at higher temperature. They also reported near “zero” residual stress values for low-temperature PEALD SiO_2_ films [21, 30]. Taking into account the residual stress value reported here and in literature, the close to “zero” stress is most likely a consequence of the intrinsic stress rather than the thermal stress. The intrinsic stress of PEALD SiO_2_ films could be then caused by the plasma effect. However, other factors such as the gas flow, the process pressure, or the used precursor cannot be ruled out [40].

## Conclusions

This work demonstrates the potential of CO_2_ as an oxidant for growing low-temperature PEALD SiO_2_ on moisture/oxygen sensitive materials. SiO_2_ films with low impurity levels and low tensile residual stress were grown at 90 °C by PEALD using CO_2_ and BTBAS as precursors. The films showed a saturated GPC of ~ 1.15 Å/cycle together with a density of ~ 2.1 g/cm^3^. This study also shows the possibility of reaching a saturated growth of the films with a very short ALD cycle time of about 4 s, which is considerably desirable for high throughput and therefore industrial applications.

## Abbreviations

ALD: Atomic layer deposition
ATR-FTIR: Attenuated total reflectance Fourier transform infrared spectroscopy
BTBAS: Bis(tertiary-butylamino)silane
GDOES: Glow-discharge optical emission spectroscopy
GPC: Growth-per-cycle
PEALD: Plasma-enhanced atomic layer deposition
PECVD: Plasma-enhanced chemical vapor deposition
PVD: Physical vapor deposition
rf: Radio frequency
TOF-ERDA: Time-of-flight elastic recoil detection analysis
XRR: X-ray reflectivity
